# Regulation of Natural Killer Cell TGF-β and AhR Signaling Pathways Via the Intestinal Microbiota is Critical for Host Defense Against Alcohol-Associated Bacterial Pneumonia

**DOI:** 10.21203/rs.3.rs-3328953/v1

**Published:** 2023-10-18

**Authors:** Derrick Samuelson, Daniel Villageliu1, Kelly Cunningham, Deandra Smith, Daren Knoell, Mason Mandolfo, Todd Wyatt

**Affiliations:** University of Nebraska Medical Center; University of Nebraska Medical Center; University of Nebraska Medical Center; University of Nebraska Medical Center; University of Nebraska Medical Center; University of Nebraska Medical Center; University of Nebraska Medical Center

**Keywords:** alcohol, aryl hydrocarbon receptor, pneumonia, microbiota, host defense, indole, TGFβ

## Abstract

Alcohol use is an independent risk factor for the development of bacterial pneumonia due, in part, to impaired mucus-facilitated clearance, macrophage phagocytosis, and recruitment of neutrophils. Alcohol consumption is also known to reduce peripheral natural killer (NK) cell numbers and compromises NK cell cytolytic activity, especially NK cells with a mature phenotype. However, the role of innate lymphocytes, such as NK cells during host defense against alcohol-associated bacterial pneumonia is essentially unknown. We have previously shown that indole supplementation mitigates increases in pulmonary bacterial burden and improves pulmonary NK cell recruitment in alcohol-fed mice, which were dependent of aryl hydrocarbon receptor (AhR) signaling. Employing a binge-on-chronic alcohol-feeding model we sought to define the role and interaction of indole and NK cells during pulmonary host defense against alcohol-associated pneumonia. We demonstrate that alcohol dysregulates NK cell effector function and pulmonary recruitment via alterations in two key signaling pathways. We found that alcohol increases transforming growth factor beta (TGF-β) signaling, while suppressing AhR signaling. We further demonstrated that NK cells isolated from alcohol-fed mice have a reduced ability to kill *Klebsiella* pneumoniae. NK cell migratory capacity to chemokines was also significantly altered by alcohol, as NK cells isolated from alcohol-fed mice exhibited preferential migration in response to CXCR3 chemokines but exhibited reduced migration in response to CCR2, CXCR4, and CX3CR1 chemokines. Together this data suggests that alcohol disrupts NK cell specific TGF-β and AhR signaling pathways leading to decreased pulmonary recruitment and cytolytic activity thereby increasing susceptibility to alcohol-associated bacterial pneumonia.

## Introduction

Alcohol use disorder (AUD) is an established risk factor for bacterial pneumonia and alcohol misuse carries a ten-fold increase in the likelihood of pneumococcal pneumonia, as well as a four-fold increase in mortality.^1, 2^ Alcohol ingestion is also associated with infection by other highly virulent respiratory pathogens including *Klebsiella pneumoniae*. Infection with *K. pneumoniae* carries a mortality almost double that of AUD patients infected by other pathogens and *K. pneumoniae* infections have historically been overrepresented in pneumonia patients with AUD.^3, 4^ A variety of mechanisms contribute to the increased risk of pneumonia in subjects with an AUD including decreased mucus-facilitated clearance of invading pathogens and pulmonary immune respones.^5^ Though the above mechanisms are well established, our research has demonstrated the importance of other novel mechanisms, which derive from alterations to the composition and function of the gut-microbiota. Precisely, we demonstrated that alcohol-associated dysbiosis increases susceptibility *Klebsiella* pneumonia, independent of alcohol consumption.^6^

Marked changes in pulmonary host defense were associated with alcohol-dysbiotic mice, including an increase in pulmonary inflammatory cytokines and a decrease in the number of immune cells (CD4 + and CD8 + T-cells) in the lung cells following *Klebsiella* infection. However, contrary to the pulmonary host response, a marked increase in immune cell counts were seen within the intestinal tract, which suggests that GI T-cell sequestration or dysregulated immune cell trafficking may impair pulmonary host defense.^6^ Similarly, we found that prophylactic treatment with indole (a microbial specific metabolite), or a cocktail of probiotics reduced both pulmonary and splenic bacterial burden, improved host immune responses, and facilitated pulmonary trafficking of immune cells. All of which were, in part, driven by aryl hydrocarbon receptors (AhR), as inhibition of AhR mitigated the protective effects. Further, indole increased the frequency of IL-22 immune cells in the lungs and small intestine, and enhanced pulmonary recruitment of CD45^+^ cells, particularly CD3^+^ T-cells and NK1.1^+^ natural killer (NK) cells.^7^

Here we demonstrate that alcohol dysregulates NK cell effector function and pulmonary recruitment via alterations in two key signaling pathways. We found that alcohol increases transforming growth factor beta (TGF-β1) signaling, while suppressing AhR signaling. We further demonstrate that NK cells isolated from alcohol-fed mice have a reduced ability to kill *Klebsiella pneumoniae*. NK cell migratory capacity to chemokines was also significantly altered by alcohol, as NK cells isolated from alcohol-fed mice exhibited preferential migration in response to CXCR3 chemokines but exhibited reduced migration in response to CCR2, CXCR4, and CX3CR1 chemokines. Together this data suggests that alcohol disrupts NK cell specific TGF-β1 and AhR signaling pathways leading to decreased pulmonary recruitment and cytolytic activity thereby increasing susceptibility to alcohol-associated bacterial pneumonia.

## Results

### NK cells are required for indole’s protective effects on pulmonary bacterial burden.

**Figure 1:**
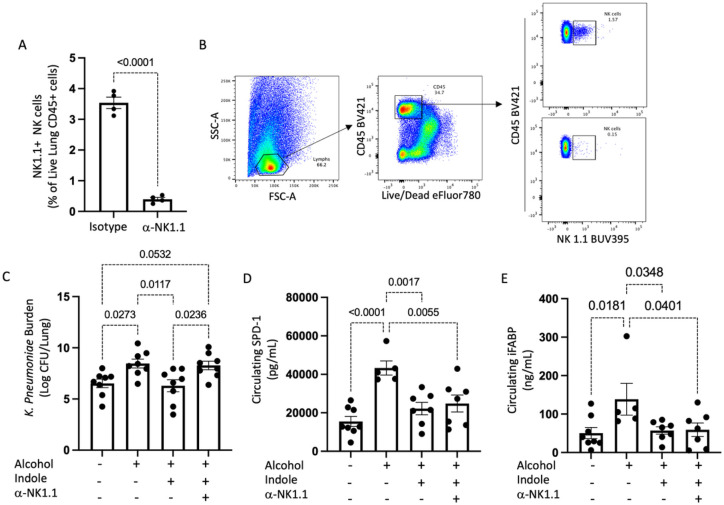
NK cells are required of optimal host defense against alcohol-associated pneumoniae. (A) Confirmation of pulmonary NK cell depletion, and (B) Representative gating strategy. (C) *Klebsiella*lung burden at 48 hrs. post infection in binge-on-chronic alcohol treated mice. (D) Circulating levels of intestinal fatty acid binding protein (iFABP) in binge-on-chronic alcohol treated mice 48 hrs. post infection. (E) Circulating levels of surfactant protein D1 (SPD-1) in binge-on-chronic alcohol treated mice 48 hrs. post infection; p values are indicated in the figure by one-way ANOVA with Sidak’s multiple comparison. N = 8/group.

Our previous data demonstrated that indole supplementation during alcohol-consumption alleviates alcohol-associated impairments in pulmonary host defense against bacterial pneumonia. Further, indole treatment restored pulmonary immune cell recruitment. However, it is unknown if indole works directly or indirectly on immune cells. Here we sought to investigate whether indole’s ability to mitigate the increased risk of alcohol-associated pneumonia was dependent on NK cells. Specifically, we selectively depleted NK cells with a monoclonal antibody against NK1.1, which lead to a greater than 95% depletion of pulmonary NK cells (Fig. 1A). Our representative gating strategy for NK cell depletion is shown in Fig. 1B. We first assessed the requirement of NK cells in indole treated mice on reducing the susceptibility to *K. pneumoniae*. Alcohol-fed mice had a higher pulmonary burden of *K. pneumoniae* 48 hrs. post infection compared to control mice (Fig. 1B). As seen previously, indole treatment reduced pulmonary *K. pneumoniae* burden in alcohol-fed mice (Fig. 1B). However, in alcohol-fed NK cell depleted mice indole treatment failed to mitigate pulmonary *K. pneumoniae* burden (Fig. 1B). Previously, we observed that indole treatment improves epithelial permeability in alcohol-fed mice yet is unknown if NK cells are involved in this process. We examined mucosal permeability following *K. pneumoniae* infection in alcohol-fed NK cell depleted mice treated with indole. Marked increases in both pulmonary and intestinal permeability were seen in alcohol-fed mice, as determined by increases in the circulating levels of surfactant protein-D (SDP-1; a biomarker of lung damage),^8^ and intestinal fatty-acid binding protein (IFABP; a biomarker of intestinal damage)^9, 10^, respectively (Fig. 1C and 1D). Alcohol-fed mice treated with indole exhibited reduced SDP-1 and IFABP levels and were indistinguishable from control fed mice after infection with *K. pneumoniae* (Fig. 1C and 1D). Interestingly, depletion of NK cells had no effect on the indole’s ability to mitigate alcohol-induced mucosal permeability (Fig. 1C and 1D). This data suggest that indole modulates host defense against bacterial pneumonia via NK cells, as loss of NK cells mitigates indoles protective effects against *K. pneumoniae*. Further, this data suggest that indole works both directly on NK cells, as well as other systems, as improvements in mucosal permeability are still preserved in NK cell depleted mice.

### Alcohol impairs NK cell migratory capacity.

**Figure 2:**
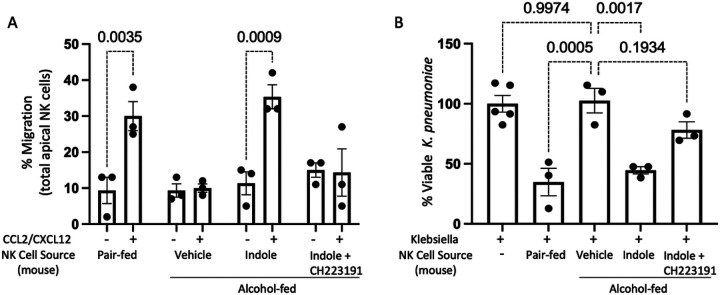
Alcohol impairs NK cell migratory capacity and bactericidal activity. (A) Mouse primary NK cells were collected from alcohol-fed and treated mice and added to the apical side of a Transwell with migration assessed in response to CCL2/CXCL12 following 5 hrs of incubation. (B) Primary NK cells were harvested from control and binge-on-chronic alcohol-fed mice with and without treatment. NK cells were then incubated with *Klebsiella* for 3 hrs. and bacterial viability was determined by serial dilution: p values as determined by one-way ANOVA with Sidak’s multiple comparison are shown in the figure. N = 3/group (6 mice per group 2 mice per NK pool).

Our previous data demonstrated that indole supplementation during alcohol-consumption restored pulmonary NK cell recruitment. However, whether this effect was due to alterations in NK cell function or changes in chemokine production in the lungs is unknown. Here we sought to characterize the functional capacity of primary NK cells isolated from pair-fed mice, alcohol-fed mice, alcohol-fed mice treated with indole, as well as mice treated with the AhR inhibitor CH223191. NK cells were purified by negative selection, allowing us to examine functional characteristics including migration and bactericidal capacity. NK cells isolated from alcohol-fed mice have a significantly reduced ability to migrate in response to the chemokines CCL2 and CXCL12 (Fig. 2A). CCL2 and CXCL12 are both strong chemo-attractants for NK cells.^11^ Conversely, primary NK cells isolated from control animals retain the ability to migrate towards CCL2 and CXCL12. Furthermore, alcohol-associated impairments in migration can be overcome by the addition of the AhR agonist indole (Fig. 2A). The effect of indole is also mediated through the AhR receptor as the addition of the AhR antagonist CH223191 blocks the effects of indole (Fig. 2A).

Additionally, NK cells isolated from alcohol-fed mice have a significantly reduced ability to kill *Klebsiella pneumoniae* in co-culture experiments (Fig. 2B). Like the migration data, primary NK cells isolated from pair-fed animals retain the ability to kill *K. pneumoniae*. The alcohol-associated impairments in bactericidal capacity were also mitigated by the addition of the AhR agonist indole (Fig. 2B). The effect of indole was likewise dependent on AhR activation, as CH223191 blocks the effects of indole on NK cell bactericidal capacity (Fig. 2B).

### Alcohol increases pulmonary and systemic TGFb levels.

**Figure 3:**
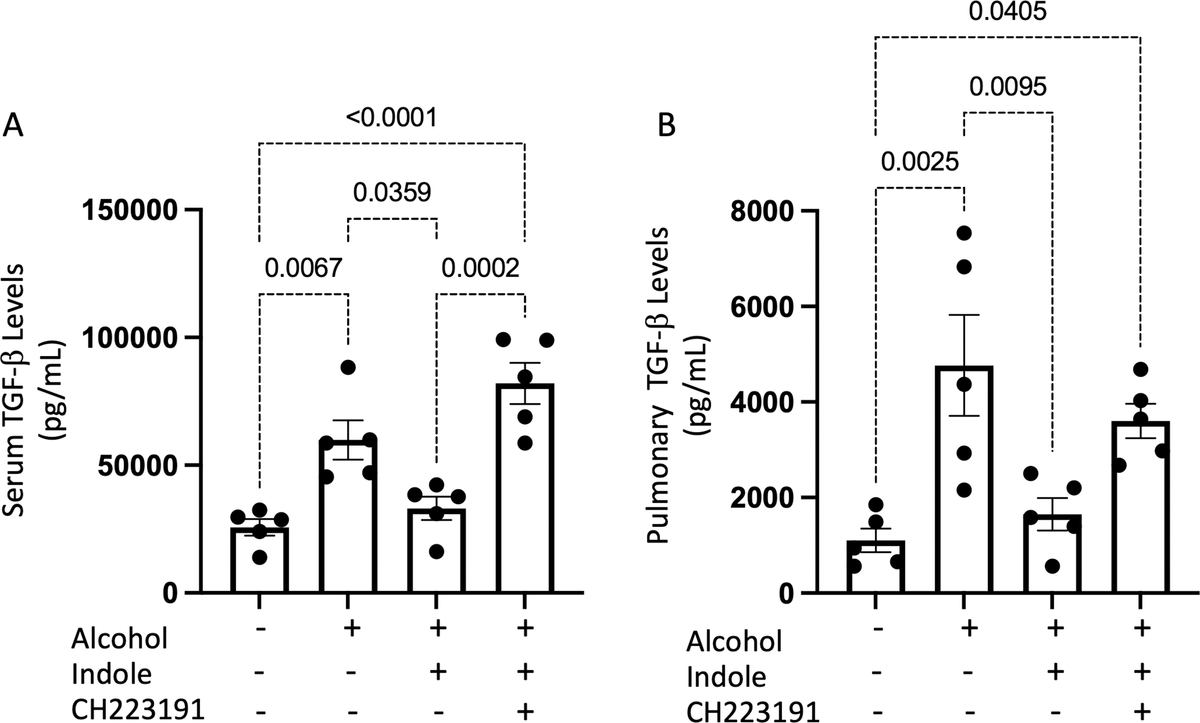
Alcohol increases pulmonary and circulating levels of TGF-b following bacterial pneumonia. (A) Pulmonary TGF-b1 levels, and (B) systemic TGF-b1 levels in binge-on-chronic alcohol-fed mice with and without *Klebsiella* infection. All graphs are 48 hrs. post infection; p values are indicated in the figure, as determined by one-way ANOVA with Sidak’s multiple comparison. N = 5/group.

The effects of alcohol on NK cell function suggests that alcohol consumption is associated with a highly immunosuppressive environment for NK cells. We sought to investigate systemic and pulmonary levels of TGF-β1, a potent immunosuppressive cytokine on NK cell function. Alcohol-fed mice exhibited marked increases in the levels of TGF-β1 in both serum and lung tissue (Fig. 3A and 3B, respectively), which is consistent with previous reports.^12^ The administration of indole suppresses the alcohol-associated increases in TGF-β1 levels which was, in part, dependent AhR activation. Specifically, in the presence of the AhR inhibitor CH223191, indole supplementation did not affect systemic or lung levels of TGF-β1 (Fig. 3A and 3B).

Additionally, we sought to evaluate the systemic and pulmonary levels of IL-22, a major cytokine downstream of AhR activation.^13^ IL-22 is also involved in wound healing and in the protection against microbial infections.^13–16^ Alcohol-fed mice exhibited marked decreases in the levels of IL-22 in both serum and lung tissue (Supplemental Fig. 1A and 1B, respectively). The administration of indole suppressed the alcohol-associated decrease in IL-22 levels and was dependent, in part, on AhR activation (Supplemental Fig. 1A and 1B).

### Manipulation of TGFb or AhR signaling pathways alters pneumonia outcomes in alcohol-fed mice.

**Figure 4:**
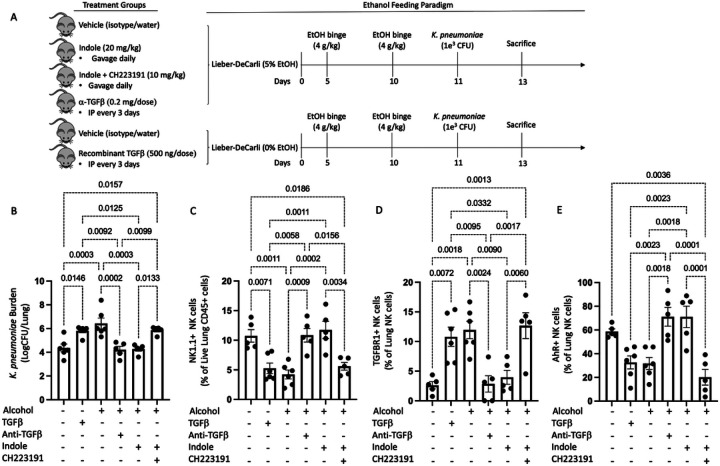
Host defense against alcohol-associated bacterial pneumonia requires activated AhR and decreased TGF-b signaling. (A) Overview of the dosing regimen for animals treated with indole, recombinant TGFβ1, anti-TGFβ1, and the AhR antagonist CH223191. (B) *Klebsiella* lung burden at 48 hrs. post infection in binge-on-chronic alcohol treated mice. Composition of CD45+ cells consisting of (C) NK1.1 cells, (D) TGFBR1+ NK cells and (E) AhR+ NK cells; p values are indicated in the figure by one-way ANOVA with Sidak’s multiple comparison. N = 5–6/group.

Divergent TGFb and AhR signaling associated with alcohol consumption suggests that alcohol use shift host defense pathways to an immunosuppressive phenotype (TGFb), while decreasing immune regulatory/stimulatory pathways (AhR). As such, we sought to pharmacologically manipulate both signaling pathways to alter the host response to bacterial infection. Specifically, cohorts of female mice were randomized into the following groups: 1) alcohol + vehicle, 2) alcohol + indole (20mg/kg), 3) alcohol + indole + CH-223191 (AhR inhibitor; 10 mg/kg), 4) alcohol + anti-TGF-b1 (10 mg/kg), 5) pair-fed + vehicle, and 6) pair-fed + TGF- b1 (0.5 μg/g) (Fig. 4A). We first assessed pulmonary bacterial burden 48 hrs post infection in all mice. Alcohol-fed mice exhibited a marked increase in pulmonary bacterial burden relative to pair-fed mice (Fig. 4B). Similarly, pair-fed mice treated with exogenous TGF-b1 or alcohol-fed mice treated with indole and the AhR inhibitor CH223191 exhibited a significant increase in pulmonary bacterial burden compared to pair-fed mice (Fig. 4B). Conversely administration of indole or the administration of anti-TGF-β1 monoclonal antibodies effectively reverses the detrimental effects of alcohol, by reducing the pulmonary bacterial burden to levels like those observed in pair-fed mice (Fig. 4B).

We also evaluated the pulmonary NK cell population following bacterial pneumonia. Our flow cytometry gating strategy for all NK cell populations is shown in Supplemental Fig. 2. Bacterial burden data for alcohol-fed mice, mice treated with the AhR inhibitor CH22319, and pair-fed mice treated with exogenous TGF-b1 exhibited a significant decrease in the percentage of pulmonary NK cells (Fig. 4C). However, indole or anti-TGF-β1 monoclonal antibody treatment effectively reverses the detrimental effects of alcohol, by increasing the percentage of pulmonary NK cells (Fig. 4C). We also found that the NK cells in the lungs of alcohol-fed mice, alcohol-fed mice treated plus indole and CH22319, as well as pair-fed mice treated with TGF-b1 expressed higher levels of TFGbR1 and lower levels of AhR (Fig. 4D, and 4E, respectively). These trends were reversed in pair-fed mice, as well as alcohol-fed mice treated with indole or the anti-TGF-β1 monoclonal antibody (Fig. 4D, and 4E).

**Figure 5:**
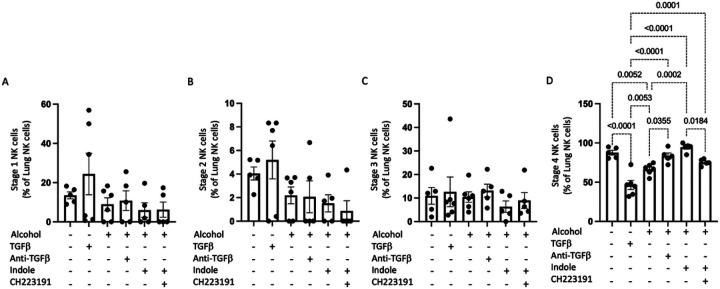
Alcohol decreases stage 4 mature pulmonary NK cells via alterations in AhR and TGFβ signaling. The proportion of pulmonary (A) stage I, (B) stage 2, (C) stage 3 and (D) stage 4 NK cells in binge-on-chronic alcohol mice treated with TGFβ1, anti-TGFβ1, indole, and CH223191; p values are indicated in the figure by one-way ANOVA with Sidak’s multiple comparison. N = 5–6/group.

NK cells were also evaluated based on the 4-stage model of maturation using CD27, and CD11b markers.^17^ Specifically, NK cells were grouped into CD11b-CD27−, CD11b-CD27+, CD11b + CD27+, and CD11b + CD27− where each step indicates the acquisition of NK cell effector functions and maturation. The percentage of stage 1, 2, or 3 NK cells were not significantly affected by alcohol consumption or by any of the exogenous treatments (Fig. 5A, 5B, and 5C). Conversely, marked effects were seen in the percentage of stage 4 NK cells in the lungs of mice post infection (Fig. 5D). Specifically, administration of either TGF-β1 or alcohol decreasing the number of stage 4 NK cells (Fig. 5D). These trends were reversed in pair-fed mice, as well as alcohol-fed mice treated with indole or the anti-TGF-β1 monoclonal antibody, as these mice exhibited a significant increase in the number of pulmonary stage 4 NK cells, compared to their respective controls (Fig. 5D).

**Figure 6:**
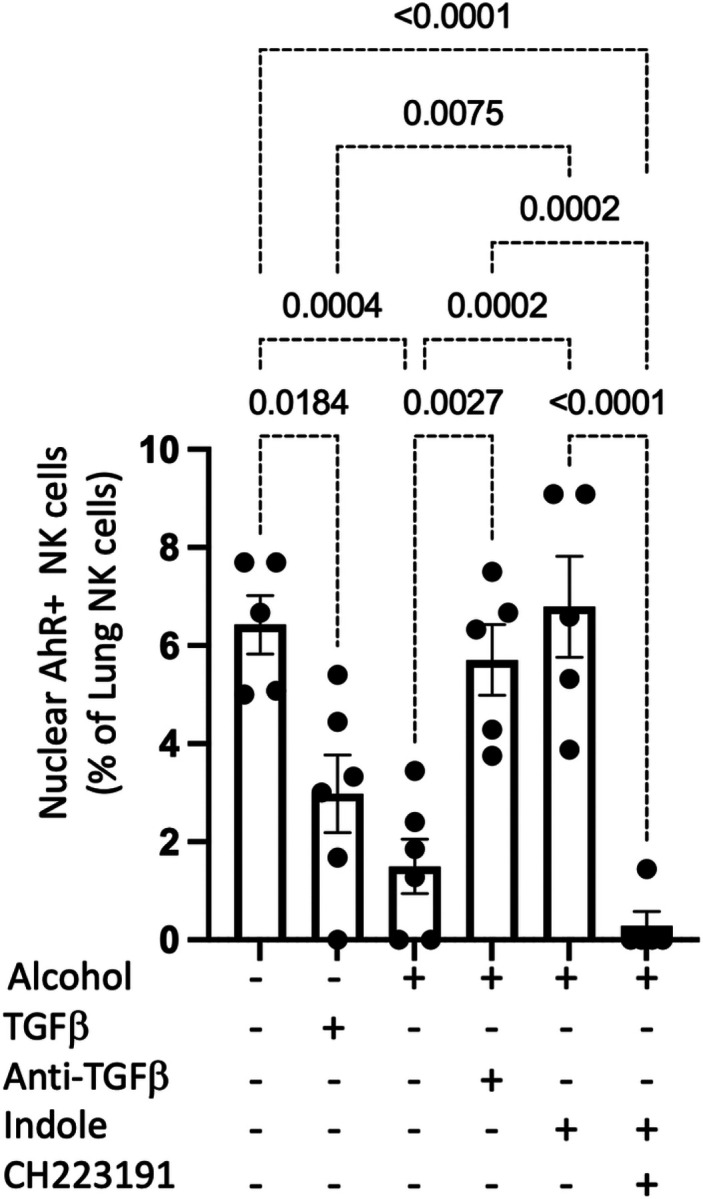
Alcohol decreases NK cells AhR nuclear translocation via alteration in AhR and TGFβ signaling. NK cells were isolated from the lungs of alcohol-fed *Klebsiella* infected mice, and the percentage of pulmonary NK cells with nuclear localization of AhR was assessed using Anims FlowSight; p values are indicated by one‐way ANOVA with Sidak’s multiple comparison. N = 5–6/group.

Finally, we evaluated the number of pulmonary NK cells with nuclear AhR (active form) using imaging flow cytometry. Administration of either exogenous recombinant TGF-β1 to control animals or alcohol decreased the number of NK cells with nuclear AhR (Fig. 6). However, pair-fed mice, as well as alcohol-fed mice treated with indole or the anti-TGF-β1 monoclonal antibody, exhibited a significant increase in the number of NK cells with nuclear AhR, compared to their respective controls (Fig. 6). Further, as expected, the AhR receptor antagonist CH223191 completely abolishes translocation of the AhR complex to the nucleus (Fig. 6).

### Alcohol and TGFb treatment increase pulmonary inflammation and epithelial leak.

**Figure 7:**
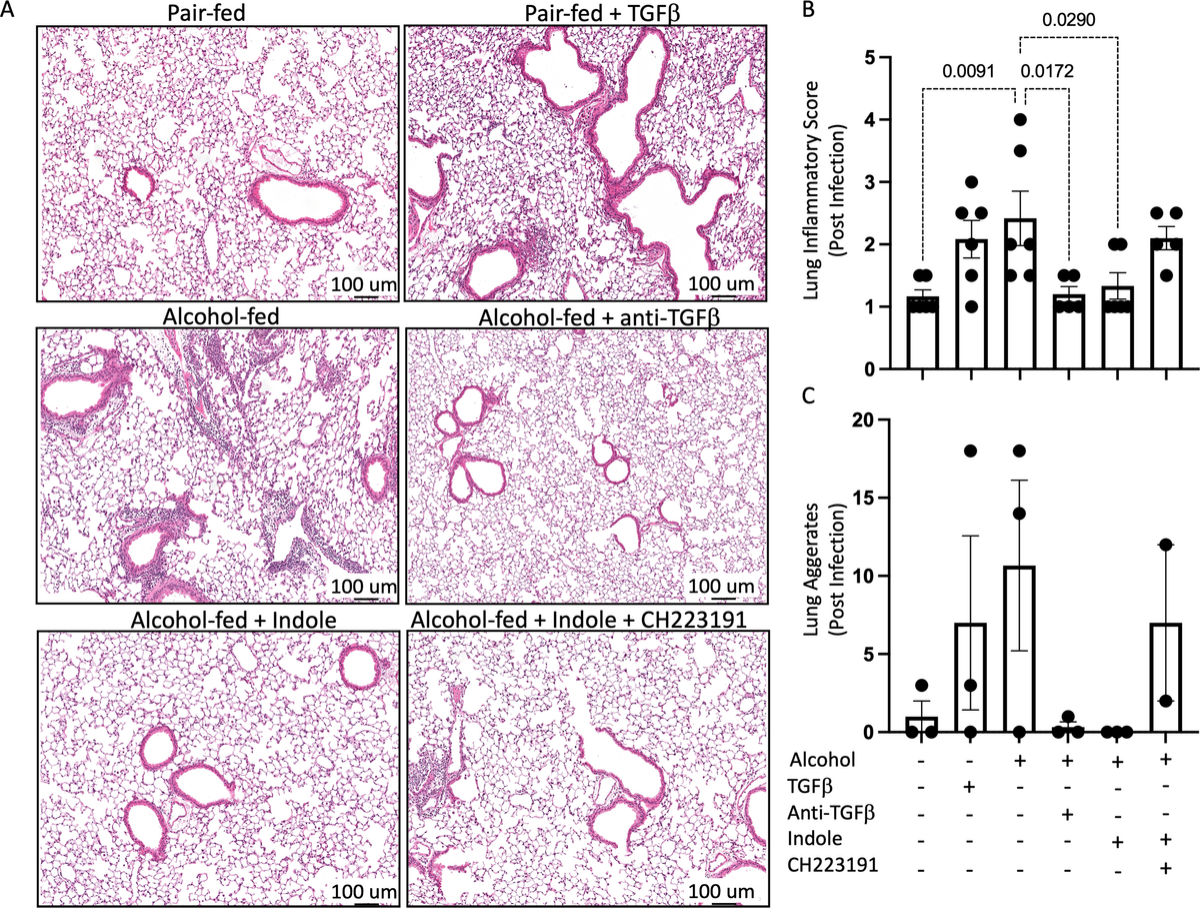
Pulmonary injury and inflammation following alcohol-associated bacterial pneumonia is mitigated by indole treatment. Alcohol-fed mice were infected with *Klebsiella*, and pulmonary damage was assessed 48 hrs. post infection. (A) Representative pulmonary histology (20x magnification) of *Klebsiella*infected mice, (B) quantification of the lung inflammatory score, and (C) lung aggregates. p values are indicated in the figure by one-way ANOVA with Sidak’s multiple comparison. N = 6/group.

Histopathologic findings were consistent with observations for pulmonary bacterial burden and immune infiltration (Fig. 7). Specifically, we observed a significant increase in inflammatory scores for mice treated with alcohol (p = 0.0091) and TGF-β1. Inflammation was alleviated to baseline by the addition of anti-TGF-β1 monoclonal antibody treatment, or indole supplementation. However, alleviation mediated by indole supplementation was not observed if indole was co-administered with the AhR inhibitor CH223191. Aggregation patterns mirrored inflammatory scores with noticeable aggregations noted for alcohol, TGF-β1 and alcohol/AhR inhibition treatments, however these findings did not reach significance (Fig. 7).

**Figure 8:**
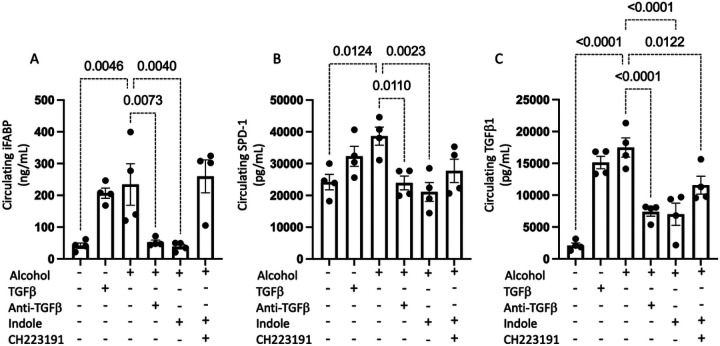
Alcohol increases epithelial barrier dysfunction and TGFb1 levels. Alcohol-fed mice were infected with *Klebsiella*, and epithelial damage was assessed 48 hrs. post infection. Circulating levels of (A) iFABP, (B) SDP-1, and (C) TGFβ1; p values are indicated in the figure by one-way ANOVA with Sidak’s multiple comparison. N = 4/group.

We observed that alcohol and TGF-β1 have several potentially detrimental effects on epithelial integrity. Consistent with our previous work, epithelial barrier function was impaired in the presence of either ethanol or TGF- β. Circulating levels of intestinal iFABP (Fig. 8A) and pulmonary SPD-1 (Fig. 8B) were increased, suggesting that barrier integrity was decreased by these treatments and contributing to immune burden via epithelial leakiness (Fig. 8). We further confirmed the effectiveness of recombinant TGF-β1 or anti-TGF-β1 monoclonal antibody treatment by measuring the levels of circulating TGF-β1. We confirmed that the exogenous administration of TGF-β1 utilized in our experiment increased TGF-β1 levels to a range like that observed in our alcohol treated animals (Fig. 8C). Interestingly, we also observed that the AhR agonist indole was able to decrease the circulating levels of TGF-β1, which could be due to improvements in epithelial integrity.

### Alcohol and TGFb treatment significantly impair NK cell function.

**Figure 9:**
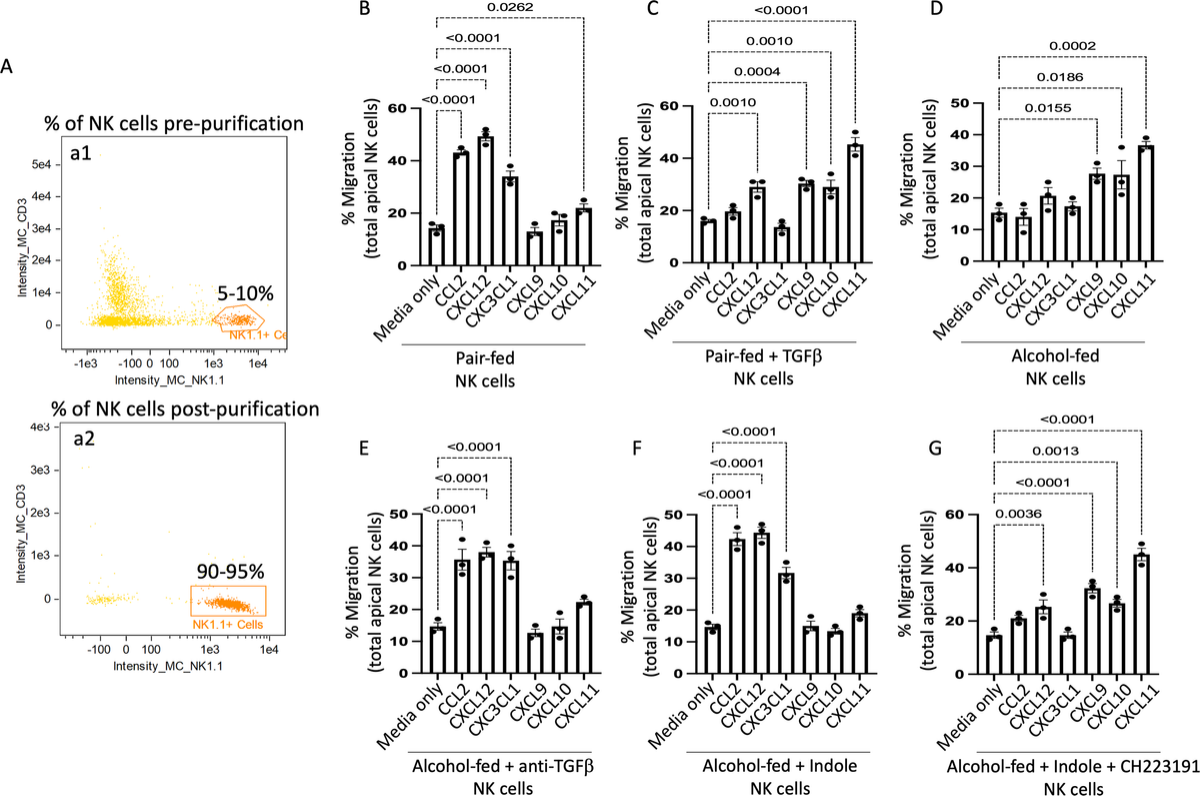
Alcohol dysregulates NK cell migration in response to chemokines. Mouse primary NK cells were collected from alcohol-fed and treated mice and added to the apical side of a Transwell and migration in response to different chemokines was assessed following 5 hrs of incubation. (A) Confirmation of NK cell purification. (a1) Percentage of NK cells pre isolation, and (a2) percentage of NK cells post isolation. NK-cell migration response profiles to different chemokines from (B) Pair-fed mice, (C) pair-fed mice treated with TGFβ1, (D) alcohol-fed mice, (E) alcohol-fed mice treated with anti-TGFβ1, (F) alcohol-fed mice treated with indole, and (G) alcohol-fed mice treated with indole and CH223191. Percent migration was calculated as the total number of viable NK cells in the bottom well divided by the total number of viable NK cells added to the Transwell insert; p values are indicated in the figure by one-way ANOVA with Sidak’s multiple comparison. N = 3/group (6 mice per group 2 mice per NK pool).

We then tested the influence of alcohol and TGF-β1 on NK cell functions including NK cell trafficking, NK bactericidal capacity, and production of antibacterial products. Specifically, circulating and splenic NK cells were isolated from the following groups of mice: 1) alcohol + vehicle, 2) alcohol + indole (20mg/kg), 3) alcohol + indole + CH-223191 (AhR inhibitor; 10 mg/kg), 4) alcohol + anti-TGF-b1 (10 mg/kg), 5) pair-fed + vehicle, and 6) pair-fed + TGF-b1 (0.5 μg/g). Circulating and splenic NK cells from two mice per treatment group (n = 3 sets of NK cells per in vivo treatment group) were pooled to generate sufficient NK cells for ex-vivo testing. NK cells were isolated via negative-selection and following purification a 90–95% pure population of NK cells was obtained (Fig. 9A). We first investigated the migratory capacity of the isolated NK cells using a Transwell migration assay. Strikingly, we observed two distinct migration response profiles to common NK cell chemokines. NK cells isolated from pair-fed mice, as well as alcohol-fed mice treated with indole, or anti-TGF-b1 monoclonal antibodies readily migrated in response to CCL2, CXCL12 and CXC3CL1 (p < 0.0001 in all cases) but were relatively non-responsive to CXCR3 signals including CXCL9, CXCL10 and CXCL11 (Fig. 9B, 9E, 9F). Conversely, we found that NK cells obtained from alcohol-fed mice or pair-fed mice treated with recombinant TGF-β1 readily migrated in response to CXCR3 signals including CXCL9, CXCL10 and CXCL11, but were non-responsive to CCL2, CXCL12 and CXC3CL1cytokines (Fig. 9C, 9D, 9G). Suggesting that alcohol use alters the NK cell chemoattractant response and migratory capacity.

**Figure 10:**
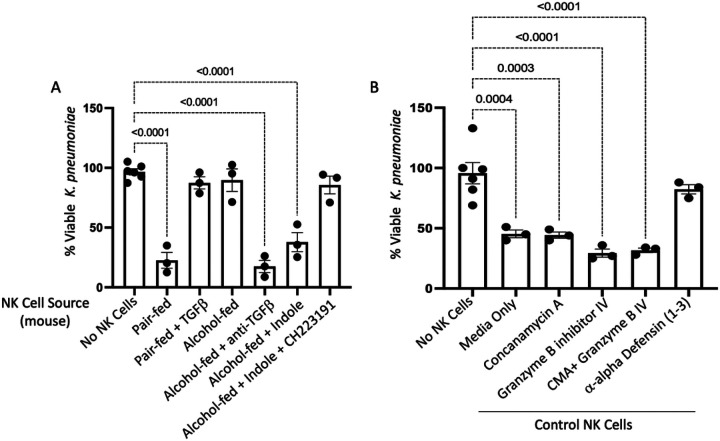
Alcohol dysregulates NK cell bactericidal capacity. Mouse primary NK cells were collected from alcohol-fed and treated mice and co-cultured with *Klebsiella* for 3 hrs. (A) Percent viable *K. pneumoniae* 3 hrs. post co-culture. Primary control NK cells were pre-treated with various inhibitors prior to co-cultured with *Klebsiella*. (B) Percent viable *K. pneumoniae* 3 hrs. post co-culture with inhibitor pre-treatment. Percent killing was calculated as the total number of viable bacteria post 3 hrs. incubation divided by number of viable bacteria grown in OptiMEM without NK cells present. p values are indicated in the figure by one-way ANOVA with Sidak’s multiple comparison. N = 3/group (6 mice per group 2 mice per NK pool).

**Figure 11:**
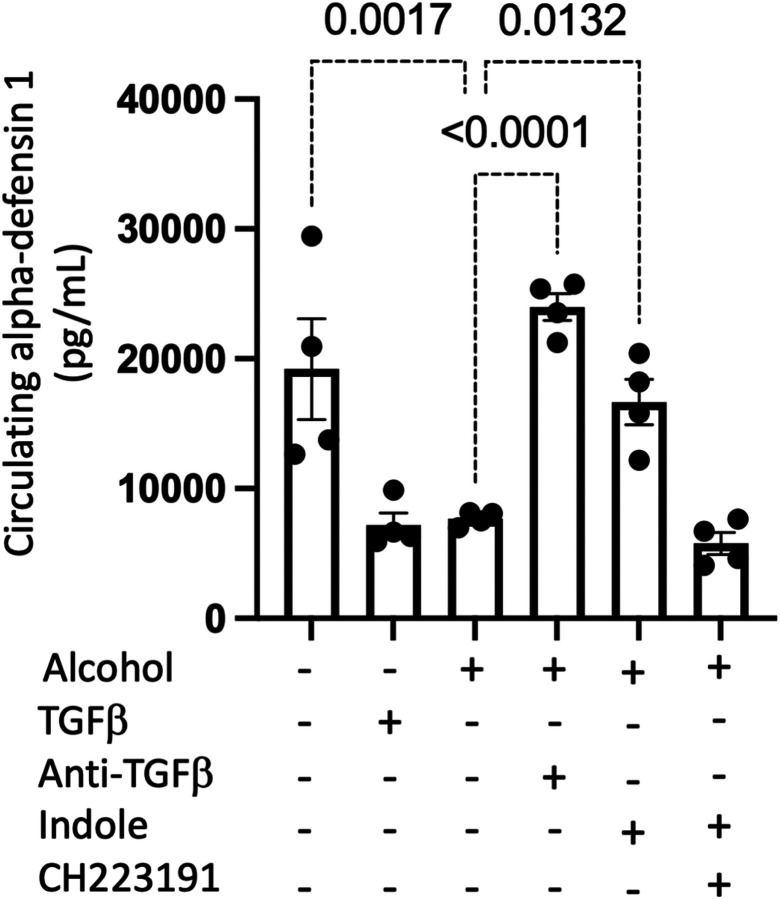
Alcohol decreases circulating alpha-defensin 1 levels. Alcohol-fed mice with and without treatments were infected with *Klebsiella* and serum was collected 48 hrs. post infection. Circulating levels of alpha-defensin 1 48 hrs. post infection; p values are indicated in the figure by one-way ANOVA with Sidak’s multiple comparison. N = 4/group.

In addition to migratory impairment, bactericidal capacity was substantially impaired in NK cells isolated from alcohol-fed mice or pair-fed mice treated with TGF-β1. NK cells isolated from pair-fed mice, as well as alcohol-fed mice treated with indole, or anti-TGF-b1 monoclonal antibodies readily suppress bacterial viability to less than 25% of that observed for bacteria grown in media over the same timeframe (Fig. 10A). However, NK cells isolated from both alcohol and exogenous TGF-β1 treatment mice exhibit a near complete abolishment of bacterial killing (Fig. 10A). The loss of the NK bactericidal function appears to be partly attributable to dysfunction in alpha-defensin related pathways. Specifically, primary NK cells isolated from control animals were pretreated with various inhibitors prior to coculture with *Klebsiella*. NK cell treatment groups included: 1) Vehicle (0.1% DMSO), 2) Granzyme B inhibitor (10,000 ng/mL), 3) Concanamycin A (100 nM), 4) Granzyme B inhibitor and Concanamycin A, and 5) anti-DEFA1 (1μg/mL) for 1 hour prior to co-culture with *Klebsiella*. Inhibition of Granzyme B or perforin did not significantly impair the bactericidal capacity of NK cells, however the inhibition of alpha-defensin greatly limited the bactericidal capacity of primary NK cells (Fig. 10B). We further validated these results with an additional bacterial pathogen (Streptococcus pneumoniae), a gram-positive organism and leading cause of alcohol-associated bacterial pneumonia. NK cell-mediated killing of S. pneumoniae was significantly impaired by alcohol and TGF-b1 and was dependent on alpha-defensin (Supplemental Fig. 3). To complement the in vitro NK cell assays in vivo measurements of circulating alpha-defensin show that both alcohol and TGF-β1 suppress circulating levels of alpha-defensin (Fig. 11). In contrast, treatment of alcohol-fed mice with indole or anti-TGF-β1 restore circulating levels alpha-defensin, overcoming the suppressive effects of alcohol (Fig. 11).

### Exogenous TGFb-1 does not exacerbate alcohol-associated pneumonia.

**Figure 12:**
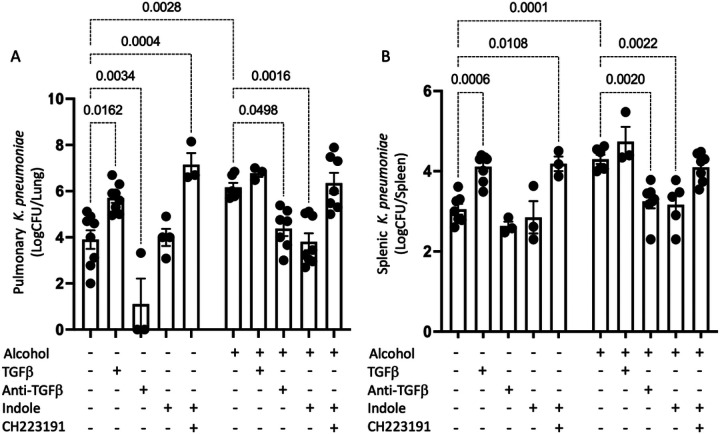
Optimal pulmonary immune responses to alcohol-associated bacterial pneumonia requires activated AhR and decreased TGF-b signaling. Pair-fed and alcohol-fed treated with indole, recombinant TGFβ1, anti-TGFβ1, and the AhR antagonist CH223191 were infected with *Klebsiella* and (A) pulmonary and (B) splenic *Klebsiella* burden was assessed 48 hrs. post infection; p values are indicated in the figure by one-way ANOVA with Sidak’s multiple comparison. N = 3–8/group.

To further validate the role of TGF-b1 and AhR signaling in host defense against alcohol-associated bacterial pneumonia we repeated the previous experimental paradigm with several additional control groups. Specifically, an additional cohort of female mice were randomization into the following groups: 1) alcohol + vehicle, 2) alcohol + indole (20mg/kg), 3) alcohol + indole + CH-223191 (AhR inhibitor; 10 mg/kg), 4) alcohol + anti-TGF-b1 (10 mg/kg), 5) alcohol + TGF- b1 (0.5 μg/g), 6) pair-fed + vehicle, 7) pair-fed + TGF- b1 (0.5 μg/g), 8) pair-fed + indole (20mg/kg), 9) pair-fed + indole + CH-223191 (10 mg/kg), and 10) pair-fed + anti-TGF- b1 (10 mg/kg). In the context of infection, we showed that effective clearance bacteria from the lungs of mice (Fig. 12A), as well as mitigation of bacterial dissemination (Fig. 12B) was dependent on a balance of increased AhR activity and decreased TGF-β1 signaling, in-line with the previous results. These data also demonstrated that exogenous TGF-β1 did not act synergistically or additively to alcohol-feeding alone.

Likewise, we isolated primary NK cells via negative selection from each of the corresponding treatment groups and assessed migratory capacity and bactericidal capacity. Similar to our previous study we found that NK cells isolated from pair-fed mice, as well as alcohol-fed mice treated with indole, or anti-TGF-b1 monoclonal antibodies readily migrated in response to CCL2, CXCL12 and CXC3CL1 but were relatively non-responsive to CXCR3 signals including CXCL9, CXCL10 and CXCL1. While the NK cells obtained from alcohol-fed mice or pair-fed mice treated with recombinant TGF-β1 readily migrated in response to CXCR3 signals including CXCL9, CXCL10 (Supplemental Fig. 4).

Finally, NK cells isolated from pair-fed mice, as well as alcohol-fed mice treated with indole, or anti-TGF-b1 monoclonal antibodies readily suppress bacterial viability, while NK cells isolated from both alcohol and exogenous TGF-β1 treatment mice exhibit abolished bacterial killing (Supplemental Fig. 5A). The loss of the NK bactericidal function appears to be partly attributable to dysfunction in alpha-defensin related pathways, as inhibition of Granzyme B or perforin did not significantly impair the bactericidal capacity of NK cells, however the inhibition of alpha-defensin greatly limited the bactericidal capacity of primary NK cells (Supplemental Fig. 5B).

### Treatment of human NK cells with exogenous alcohol or TGFb significantly impair NK cell function.

**Figure 13:**
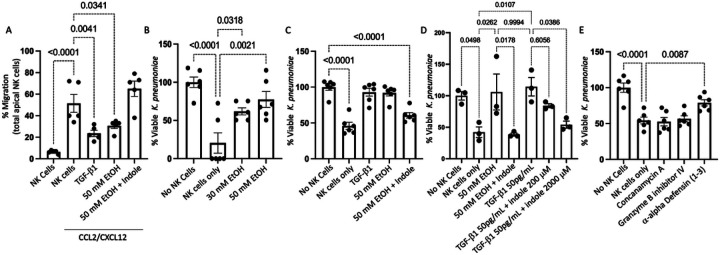
Alcohol dysregulates human NK cell migration and bactericidal capacity. **Primary human** NK cells (NK-92 line) were treated with alcohol, indole, or recombinant TGFβ1 and NK cell function was assessed. (A) NK cell migration in response to the CCL2/CXCL12. (B-E) Percent viable *K. pneumoniae* 3 hrs. post co-culture with NK cells pre-treated with different compounds/inhibitors. (B) Ethanol dose dependent inhibition of bactericidal capacity. (C) Inhibition of bactericidal capacity via TGFβ1 and rescue of alcohol-mediated inhibition via indole. (D). Indole improves TGFβ1 mediated suppression of bactericidal capacity in a dose dependent manner. (E) NK cell bactericidal capacity mediated via alpha-defensin; p values are indicated in the figure by one-way ANOVA with Sidak’s multiple comparison. N = 3–6/group.

We then tested the influence of exogenous alcohol and TGF-β1 on NK cell function using a human NK cell line (NK-92 cells). Specifically, NK-92 cells were pre-treated with 50 mM EtOH, 50 pg/mL of human TGF-b1, or with 20 μM indole and 50 mM EtOH for 24 hours. Following incubation NK cell migratory capacity was assessed using the Transwell migration assay. NK-92 cell treated with either EtOH alone or with TGF-b1failed to migrate in response to CCL2/CXCL12, compared to untreated NK-92 cells (Fig. 13A). Further, alcohol-treated NK-92 cells which also were supplemented with exogenous indole had improved migratory capacity in response to CCL2/CXCL12, compared to alcohol treated NK-92 cells (Fig. 13A).

In addition to migratory impairment, bactericidal capacity was substantially impaired in NK-92 cells pretreated with alcohol. Specifically, NK-92 cells treated with EtOH for 24 hours prior to co-culture with *Klebsiella* exhibited a dose dependent decrease in bactericidal capacity, compared to untreated NK-92 cells. Additionally, NK-92 cells pre-treated with 50 mM EtOH or 50 pg/mL of human TGF-b1, while alcohol-treated NK-92 cells also supplemented with exogenous indole had improved NK cells bactericidal capacity (Fig. 13C). Finally, the effects of TGF-b1 suppression of NK-92 cells bactericidal capacity could be mitigated by exogenous indole treatment in a dose dependent manner (Fig. 13D). However, the required dose of indole to mitigate TGF-b1 suppression was 100 times that required to overcome the effects of alcohol alone. Like our mouse *ex-vivo* data, loss of the NK-92 bactericidal function appears to be partly due to impaired alpha-defensin production, as the inhibition of Granzyme B or perforin did not significantly impair the bactericidal capacity of NK-92 cells, but the inhibition of alpha-defensin greatly limited the bactericidal capacity of NK-92 cells (Fig. 13E).

## Discussion

Numerous preclinical and clinical studies have demonstrated the critical role intestinal microbiota play in regulating and facilitating pulmonary host defense against bacterial and viral infections.^6, 18–24^ Mice devoid of GI microbiota (germ-free mice) or mice with significantly depleted microbial communities (antibiotic-treated mice) are highly susceptible to pulmonary infection with various bacterial and viral pathogens, including *K. pneumoniae* and S. pneumoniae.^18, 19^ Several mechanistic pathways have been identified that regulate the gut-lung axis. Specifically, Nod-like receptor-stimulating bacteria present in the GI tract were shown to increase levels of interleukin-17A, which lead to the production of granulocyte–macrophage colony-stimulating factor and killing of bacterial pathogen by alveolar macrophages.^19^ Enhanced susceptibility to *K. pneumoniae* in germ-free mice was also associated increased levels of IL-10, decreased neutrophil pulmonary recruitment, and bacterial growth and dissemination.^18^ However, the rigor of prior research linking alcohol dysbiosis to bacterial pneumonia is limited. In fact, to our knowledge, we are the only group to have investigated the role of alcohol-associated intestinal dysbiosis on pulmonary infections.^6, 25^ In addition, nothing is known about how alcohol might influence NK cell recruitment to the lungs.

NK cells have been classically viewed as crucial for innate defense against viruses and intracellular bacteria,^26, 27^ however emerging data indicate that NK cells also participate and are critical for optimal host defense against extracellular bacteria.^28^ For example, it has been shown in mouse models that NK cells are required for optimal pulmonary host defense against Pseudomonas aeruginosa,^30^ and Staphylococcus aureus.^29^ Similarly, NK cell are also required to combat infection of the GI tract with the extracellular bacterium Citrobacter rodentium.^31^ In addition, lung NK cells promote host defense against respiratory infection by *K. pneumoniae* through the production of IL-22 and IFN-γ.^14, 32^ The mechanisms whereby NK cells protect against bacterial infections remain ill-defined, but the production of cytokines, such as TNF-α, IFN-γ, IL-22, and IL-10, the recruitment of additional leukocytes, stimulation of macrophages, and direct bacterial killing are likely key mechanistic factors. Alcohol misuse is known to quantitatively and qualitatively alter NK cell function.^33^ Specifically, alcohol reduces peripheral NK cell numbers, increases the number of IFN-γ producing NK cells, and compromises NK cell cytolytic activity by inhibiting the production of perforin, granzyme A, and granzyme B.^34–36^

Recently, AhR signaling has emerged as a potential link between the intestinal microbiota and the host immune system, especially regarding host defense against pathogenic insult. For example, AhR-dependent expression of IL-22 is shown to be critical for host defense against *Candida albicans*.^13^ Additionally, mice treated with antibiotics exhibited marked impairments in lung immunity to *P. aeruginosa* via decreased AhR expression in the intestinal tract and reduced peroyxnitrite production by AMs. Importantly, augmentation of AhR signaling by tryptophan supplementation (catabolized by the microbiota to AhR ligands) decreases *P. aeruginosa* bacterial counts in the lung and increases intestinal ROS production, as well as phagocytic activity of AMs.^37^ AhR signaling was first demonstrated to be important for the development and differentiation of regulatory and Th17 T-cells.^38^ Since then, AhR expression has been reported in specialized innate lymphoid cells,^39^ as well as in a number of other hematopoietic populations. Recently, NK cells were found to express AhR upon cytokine stimulation.^40–42^ Interestingly, determinants of AhR activity, including AhR ligands found in the diet, were found to modulate the antitumor effector functions of NK cells.^41^ Similarly, the activity of NK cells is strongly affected by TGF-β. Specifically, TGF-β is known to: 1) negatively regulate IFN-γ production,^43^ 2) decrease the surface level of the activating receptors NKG2D and NKp30,^44^ 3) decrease cytotoxicity,^45^ and 4) decrease antitumor function of NK cells.^45^ TGF-β is also known to alter the surface expression of the chemokine receptors CXCR3, CXCR4, CX3CR1, which possibly impacts NK cell migration and recruitment.^44^

**Figure 14:**
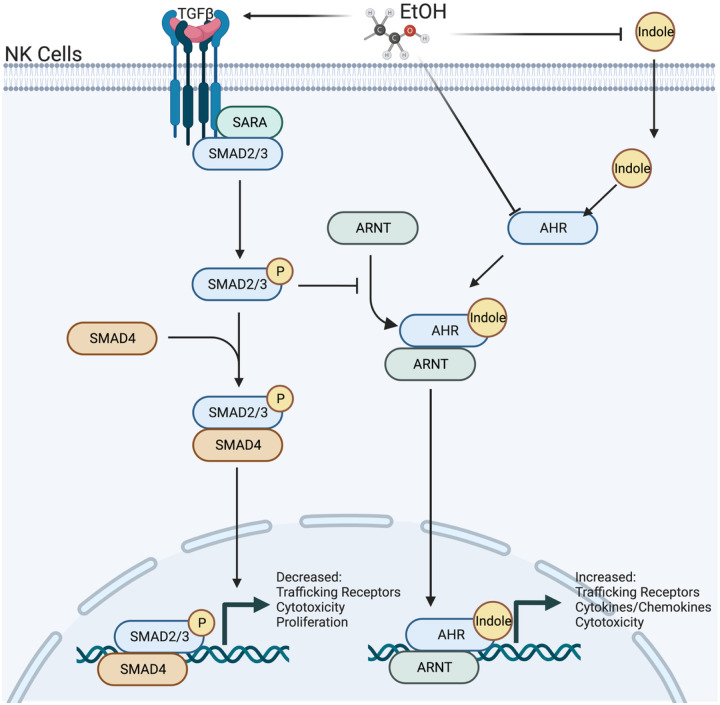
Working model of alcohol’s effects on TGFβ and AhR signaling in NK cells. Alcohol-associated microbiota increases systemic TGF-b1 and decreases AhR signaling in NK cells leading to impaired function and trafficking to the lungs in response to infection. Image created with BioRender.com.

We propose that indole and TGF-b1 exert counter-regulatory effects through differential genetic expression regulated by AhR and SMAD2/3, respectively. Our working model is shown in Fig. 14. The experiments above establish that AhR and TGF-β1 interplay is relevant for a variety of immunological processes including circulating levels of alpha-defensins, NK cellular migration, and NK cytotoxicity, as well as epithelial barrier function. In addition, our data suggests that alcohol may likewise interfere with NK cell maturation through changes in AhR and SMAD2/3 signaling, although lineage tracking experiments are needed to confirm/validate these observations. Exposure to alcohol, through TGF-β signaling, alters the functional effectiveness and phenotypic characteristics of pulmonary NK cells. However, whether this represents a change in gene expression of circulating pools of NK cells or in the maturation of NK cells from stage 1 to stage 4 cells is unknown. We currently favor the later explanation and contend that SMAD2/3 and AhR signaling are opposing forces which control whether NK cells shift from a “normal” response profile to an “alcohol-associated” response profile. More precisely, following pulmonary infection, NK cells with a “normal” profile robustly respond to chemokines, such as CCL12, CXCL12, CXC3L1, while the phenotype of alcohol exposed NK cells inverts to respond preferentially to CXCL9–11 chemokines.

This study focused on the intestinal microbiota from alcohol consuming mice to understand the relationship between alcohol, the microbiota, TGF-b and AhR signaling in NK cells, and host defense against bacterial pneumonia. Together this data suggests that alcohol causes disruption of NK cell-specific TGF-β and AhR signaling leading to decreased pulmonary recruitment and cytolytic activity thereby increasing susceptibility to alcohol-associated bacterial pneumonia. Understanding the role of intestinal dysbiosis and TGF-β/AhR signaling pathways in the context of alcohol-associated pneumonia may foster novel prophylactic strategies (i.e., indole treatment) for the prevention of alcohol-associated pneumonia in high-risk individuals, which could be especially impactful for individuals with limited resources and access to alcohol treatment centers.

## Methods

### Mice.

Female 10–12-week-old C57BL/6 mice were obtained from Charles Rivers Breeding Laboratories (Wilmington, MA) and maintained in Comparative Medicine at UNMC. Food and water were provided ad libitum. All mice were housed in a SPF environment under standard social housing conditions with appropriate environmental enrichment. All protocols used in these studies were approved by the Institutional Animal Care and Use Committee of UNMC (IACUC# 19-120-11-EP). This research protocol is accordance with the NIH and Office of Laboratory Animal Welfare (OLAW) guidelines.

### Alcohol feeding model.

We utilized a binge-on-chronic alcohol feeding model as we have previously published.^6^ All animals were acclimated to liquid diet for 5 days using Lieber-DeCarli ‘82 Shake and Pour (Bioserv, Flemington, NJ. Cat. No. F1259). Groups of mice (n = 3 per cage) were randomized into ethanol-fed (Bioserv. Cat. No. F1258), ethanol-fed plus treatment, or pair-fed groups. Pair-fed mice were maintained on control-liquid diet adjusted daily according to the consumption of ethanol-fed mice. Mice were administered 4 g kg^−1^ (31.5% vol/vol) ethanol by gavage following 5 and 10 days of alcohol-feeding. Pair-fed control mice were gavaged with 7 g kg^−1^ (45% wt/vol) maltose dextrin on days 5 and 10 of control-diet feeding.

### Exogenous treatments (Indole, CH223191, anti-TGF-b1, and TGF-b1 treatment).

Mice were treated by oral gavage daily throughout the course of the experiments with indole or CH-223191. Mice received indole by gavage (20 mg/kg/dose indole: Sigma Aldrich, St. Louis, MO. Cat. No. BP0057) dissolved in sterile water warmed to 55°C. Mice received CH-223191 by gavage (10 mg/kg/dose or ~ 200 μL of 3 mM CH-223191: MedChemExpress, Monmouth Junction, NJ. Cat. No. HY-12684) dissolved in sterile corn oil. Mice were treated via IP injection with recombinant TGF-b1 or anti-TGF-b1 every 3 days with the first dose coinciding with the initiation of alcohol-feeding. Mice were treated with mouse InVivoPlus anti-TGF-b1 monoclonal antibody (clone: 1D11.16.8) by IP injection (10 mg/kg/dose, of mouse anti-TGF-b1; BioXCell, Lebanon, NH. Cat. No. BP0057). Mice were treated with mouse recombinant TGF-b1 by IP injection (0.5 μg/g/dose of rTGF-b1: MedChemExpress. Cat. No. HY-P7117). All mice not receiving treatment via oral gavage were also gavaged daily with vehicle (PBS). Similarly, animals not receiving IP treatments were IP injected with a mouse IgG1 isotype control antibody (clone MOPC-21) in PBS (10 mg/kg/dose, of mouse IgG1 isotype control: BioXCell. Cat. No. BE0083).

### NK cell depletion.

Mice were depleted of NK cells via IP injection with anti-NK1.1 monoclonal antibody every 3 days with the first dose coinciding with the initiation of alcohol-feeding. Mice were treated with InVivoPlus mouse anti-NK1.1 (clone: PK136) monoclonal antibody by IP injection (10 mg/kg/dose, of mouse anti-NK1.1; BioXCell. Cat. No. BP0036). All control mice were given IP injected with a, mouse IgG1 isotype control antibody (clone MOPC-21) in PBS (10 mg/kg/dose, of mouse IgG1 isotype control: BioXCell. Cat. No. BE0083).

### Klebsiella pneumoniae infection.

*Klebsiella* pneumoniae infections and burden assessments were performed using standard methods.^6^ Briefly, *K. pneumoniae* (Strain 43816, serotype 2; American Type Culture Collection [ATCC], Manassas, VA. Cat. No. 43816) was grown in 100 mL tryptic soy broth (Becton Dickinson, Franklin Lakes, NJ) in a shaking incubator at 37°C for 18 hours. Bacteria were then pelleted and resuspended in PBS at an estimated concentration of 1 × 10^3^ colony-forming units (CFU)/mL. Mice were then infected with 100 μl of inoculum (1 × 10^2^ CFU) via oropharyngeal aspiration. Mice were sacrificed 48 hrs. post infection, and pulmonary and splenic burden was determined via serial dilution and plating onto HiCrome *Klebsiella* Selective Agar plates.

### Lung Histology.

Whole lungs were inflated with 10% formalin (ThermoFisher Scientific) to maintain pulmonary structure. The UNMC Tissue Sciences Core Facility processed all the lungs. Specifically, lungs were paraffin embedded and 4–5 μm sectioned were mounted on glass slides and stained with hematoxylin and eosin. The Leica Aperio CS2 (Leica Biosystems, Deer Park, IL) imaging system and the Leica ImageScope software was used to obtain all images (20x). Lung inflammation was scored by a blinded pathologist using a previously published scoring system.^46, 47^

### Serum Analysis.

Serum was collected from all mice at sacrifice using BD serum separator tubes (BD Biosciences, San Jose, CA). Mouse serum levels of SPD-1 were detected via the Mouse SP-D Quantikine ELISA Kit (Novus Biologicals, Centennial, CO. Cat. No. MSFPD0). Mouse serum levels of IFABP were detected using the Mouse FABP2/I-FABP ELISA Kit (Novus Biologicals. Cat. No. NBP2–82214). Mouse alpha-defensin levels were detected using the Mouse Defensin Alpha 1ELISA Kit (LS Bio, Shirley, MA. Cat. No. LS-F6926-1). Mouse TGF-b1 levels were determined using the Mouse TGF-beta 1 DuoSet ELISA (R&D Systems, Minneapolis, MN. Cat. No. DY1679). Mouse IL-22 levels were determined using the Mouse/Rat IL-22 Quantikine ELISA Kit (R&D Systems. Cat. No. M2200). ELISA Kits were run according to manufacturer’s specifications.

### Flow Cytometry.

Lungs immune cells were collected for flow cytometry analysis.^6^ Lung tissue of each animal were collected into C-tubes and immune cells were isolated using the mouse Lung Dissociation Kit (Miltenyi Biotec, Auburn, CA. Cat. No. 130-095-927) according to manufacturer’s specifications. Lung samples were homogenized using gentleMACS^™^ Octo Dissociator (Miltenyi Biotec). Following isolation any remaining red blood cells (RBCs) were lysed using RBC lysis buffer (BioLegend, San Diego, CA. Cat. No. 420301) prior to staining. After washing with PBS, viable cells were counted on a hemocytometer using the trypan blue–exclusion method. One million viable cells were then used for staining. Lung immune cells were pretreated with TruStain FcX Anti-mouse CD16/32 antibody (BioLegend. 2 μL per test, Cat. No. 101320) and then stained with LIVE/DEAD^™^ Fixable Violet Dead Cell Stain Kit (ThermoFisher Scientific, Waltham, MA. 1 μL/test, Cat. No. L34955, Invitrogen, Eugene, OR) followed by surface staining with antibodies specific for murine CD45 Brilliant Violet 510 (BioLegend. 0.50 μg per test, Cat. No. 103137), CD3 PE-Cyanine5 (BioLegend. 0.30 μg per test, Cat. No. 100274), NK1.1 PE-eFluor^™^ 610 (ThermoFisher Scientific. 0.50 μg per test, Cat. No. 61-5941-82), CD11b APC-Cyanine7 (BioLegend. 0.60 μg per test, Cat. No. 101226), CD27 APC (BioLegend. 0.20 μg per test, Cat. No. 124212), TGFbR1 PE (ThermoFisher Scientific. 10 μL per test, Cat. No. FAB5871P100). Cells were then fixed and permeabilized using the True-Nuclear^™^ Transcription Factor Buffer Set, according to manufactures’ specifications (BioLegend. Cat. No. 424401). Cells were then stained for the intracellular markers AhR Alexa Fluor^®^ 488 (BD Biosciences, Franklin Lakes, NJ. 0.20 μg per test, Cat. No. 565788), and the nuclear stain DRAQ5 (BD Biosciences. 1.0 μL of a 1/1000 dilution of the stock per test, Cat. No. 564903). All samples were stained in the presence of BD Horizon^™^ Brilliant Stain Buffer (BD Biosciences. 50 μL per test, Cat. No. 100274). For all experiments, cells were acquired using an Amnis^®^ FlowSight^®^ Imaging Flow Cytometer (Millipore Sigma, Burlington, MA). Cells were analyzed using the IDEAS^®^ Software package (Millipore Sigma).

### NK cell isolation.

Spleen were harvested at sacrifice, resuspended in PBS, and homogenized using gentleMACS^™^ Octo Dissociator (Miltenyi Biotec). Whole blood was collected using BD Microtainer^®^ Blood Collection Tubes with K2EDTA (BD Biosciences). Splenic homogenates and whole blood were then combined and red blood cells (RBCs) were lysed using RBC lysis buffer (BioLegend, Cat. No. 420301). NK cells were then isolated using the NK Cell Isolation Kit, mouse (Miltenyi Biotec, Cat. No. 130-115-818), according to manufactures’ recommendations. Isolated NK cells were then cultured in NK MACS Medium (Miltenyi Biotec, Cat. No. 130-114-429) containing 5% Horse Serum (ThermoFisher. Cat. No. 16050130), 100 U/mL and 100 μg/mL of penicillin/streptomycin (Sigma. Cat. No. P4333-100ML), and 500 IU/mL of mouse IL-2 (ThermoFisher. Cat. No. PMC0025) for 24 hours at 37 °C with 5% CO_2_. Following 24 hours of incubation NK cells migration and bactericidal activity was assessed, described below.

### Human NK cells.

Human NK-92 cells were obtained from (American Type Culture Collection. Cat. No. CRL-2407). NK-92 cells were cultured in NK MACS Medium (Miltenyi Biotec, Cat. No. 130-114-429) containing 5% Horse Serum (ThermoFisher. Cat. No. 16050130), 100 U/mL and 100 ug/mL of penicillin/streptomycin (Sigma. Cat. No. P4333-100ML), and 500 IU/mL of human IL-2 (ThermoFisher. Cat. No. PMC0025). NK-92 cells were incubated at 37 °C with 5% CO_2_. Cells were maintained at a density of ~ 2 × 10^5^ viable cells/mL and subcultured every 2–3 days depending on density.

### NK cell migration assay.

#### Primary mouse NK cells.

NK cell migration was assessed using Corning^®^ Transwell^®^ polycarbonate membrane cell culture inserts (Sigma, Cat. No. CLS3421-48EA). The number of viable Primary NK cells was determined following 24 hours of culture via trypan blue staining. NK cells were centrifuged at 300 × ~ g for 10 minutes and resuspended in fresh OptiMEM at a density of 1 × 10^7^ viable cells/mL, and 100 μL of the NK cell suspension was added to the Transwell insert. 600 μL OptiMEM only, or OptiMEM containing 50 ng/mL of CCL2 (Sigma. Cat. No. 45-SRP3215-10UG), 100 ng/mL of CXCL12 (R&D Systems, Cat. No. 460-SD-050), 100 ng/mL of CXCL9 (R&D Systems, Cat. No. 492-MM-010), 100 ng/mL CXCL10 (R&D Systems, Cat. No. 466-CR-010), 100 ng/mL CXCL11 (R&D Systems, Cat. No. 572-MC-025), 300 ng/mL CX3CL1 (R&D Systems, Cat. No. 472-FF-025) was added to the bottom of the of the Transwell. Migration was then assessed by determining the number of viable cells in the bottom culture well 5 hours post incubation. Percent migration was calculated as the total number of viable NK cells in the bottom well divided by the total number of viable NK cells added to the Transwell insert.

#### Human NK-92 cells.

NK-92 cell migration was assessed as described for primary mouse NK cells, with the following modifications. NK-92 cells were first pretreated with 50 pg/mL of human recombinant TGFb1 (R&D Systems, Cat. No. 7754-BH-025/CF), 50 mM EtOH, or 50 mM EtOH with 20 μM indole for 24 hours. Following pre-treatment NK cells were centrifuged at 300 × ~ g for 10 minutes and resuspended in fresh OptiMEM at a density of 1 × 10^7^ viable cells/mL, and 100 μL of the NK cell suspension was added to the Transwell insert. 600 μL OptiMEM only, or OptiMEM containing 50 ng/mL of CCL2 (R&D Systems. Cat. No. 279-MC-050/CF) and 100 ng/mL of CXCL12 (R&D Systems, Cat. No. 350-NS-050/CF) was added to the bottom of the Transwell. Migration was then assessed by determining the number of viable cells in the bottom culture well 5 hours post incubation.

### NK cell bactericidal activity.

#### Primary mouse NK cells.

NK cell bactericidal capacity was assessed by co-culturing NK cells with *Klebsiella* pneumoniae (ATCC, Cat. No. 43816) or *Streptococcus pneumoniae* (Strain TIGR4 [JNR.7/87], serotype 4; ATCC, Cat. No. BAA0334). Primary NK cells isolated from each of the mouse treatment groups were centrifuged at 300 × ~ g for 10 minutes and resuspended in fresh OptiMEM at a density of 1 × 10^6^ viable cells/mL. 100 μL of the primary NK cells were added to 900 μL (1 × 10^5^ cells/well) of OptiMEM. In addition, a separate set of primary NK cells isolated from control mice was added to OptiMEM containing 10,000 ng/mL of Granzyme B inhibitor (Sigma, Cat. No. 368056), 100 nM of Concanamycin A (Sigma, Cat. No. C9705), or 1μg/mL of anti-DEFA1 (Biorbyt, Cambridge, United Kingdom. Cat. No. orb10527) for 1 hour prior to co-culture with bacteria. *Klebsiella* was grown in 100 mL tryptic soy broth (BD Biosciences) in a shaking incubator at 37°C for 18 hours. Bacteria were then pelleted and resuspended in PBS at an estimated concentration of 1 × 10^7^ colony-forming units (CFU)/mL. S. pneumoniae was grown in 100 mL Todd Hewitt Broth (BD Biosciences) at 37°C in 5% CO_2_ for 6 hours. Bacteria were then pelleted and resuspended in PBS at a concentration of 1 × 10^7^ CFU/mL. Bactericidal activity was then assessed by adding 100 μL (MOI of 10) of either *Klebsiella* or *Streptococcus* to the wells containing NK cells and incubated for 3 hours. The number of viable *Klebsiella* and *Streptococcus* 3 hours post infection was determined via serial dilution and plating onto BD BBL^™^ Trypticase^™^ Soy Agar (TSA II^™^) with sheep blood (BD Biosciences). Percent killing was calculated as the total number of viable bacteria post 3 hrs. incubation divided by number of viable bacteria grown in OptiMEM without NK cells present.

#### Human NK-92 cells.

NK-92 cell bactericidal activity was assessed as described for primary mouse NK cells, with the following modifications. NK-92 cells were first pretreated with 50 pg/mL of human recombinant TGFb1 (R&D Systems, Cat. No. 7754-BH-025/CF), 30 mM EtOH, 50 mM EtOH, 50 mM EtOH with 20 μM indole, and 50 pg/mL of human recombinant TGFb1 in the presence of 200, and 2000 μM indole for 24 hours. In addition, a separate set experiments with NK-92 cells, cells were pretreated with 10,000 ng/mL of Granzyme B inhibitor (Sigma, Cat. No. 368056), 100 nM of Concanamycin A (Sigma, Cat. No. C9705), or 1μg/mL of anti-human DEFA1–3 (Alpha Diagnostic International, San Antonio, TX. Cat. No. HDEFA11-A) for 1 hour prior to co-culture with bacteria. Bactericidal activity was then assessed as descried above.

## Statistics and Reproducibility

Statistical analyses were performed using GraphPad Prism 10 (La Jolla, CA). Results are shown as the mean ± standard error of the mean. A p < 0.05 was deemed significant. Sample size and number of replicates is indicated in each respective figure legend. Statistical significance was assessed using a Mann-Whitney test for comparisons between two groups and a one-way analysis of variance (ANOVA) with Sidak’s multiple comparison test for comparisons between three or more groups.

## Data Availability

All the data that support the findings of this study are available within the paper and are available at https://doi.org/10.6084/m9.figshare.24050628.v1.
